# Transcutaneous Electrical Nerve Stimulation Integrated into Pants for the Relief of Postoperative Pain in Hip Surgery Patients: A Randomized Trial

**DOI:** 10.1155/2024/6866549

**Published:** 2024-06-27

**Authors:** Yohann Opolka, Courage Sundberg, Robin Juthberg, Amelie Olesen, Li Guo, Nils-Krister Persson, Paul W. Ackermann

**Affiliations:** ^1^ Polymeric E-Textile Research Group Swedish School of Textiles University of Borås, Allégatan 1, Borås 501 90, Sweden; ^2^ Karolinska University Hospital, Solna, Stockholm 171 76, Sweden; ^3^ Department of Molecular Medicine and Surgery Karolinska Institutet, Solna, Stockholm 171 76, Sweden; ^4^ Smart Textiles Science Park Borås, Allégatan 1, Borås 501 90, Sweden

## Abstract

**Background:**

The effect of transcutaneous electrical nerve stimulation (TENS) on pain and impression of change was assessed during a 2.5-hour intervention on the first postoperative days following hip surgery in a randomized, single-blinded, placebo-controlled trial involving 30 patients.

**Methods:**

Mixed-frequency TENS (2 Hz/80 Hz) was administered using specially designed pants integrating modular textile electrodes to facilitate stimulation both at rest and during activity. The treatment outcome was assessed by self-reported pain Numerical Rating Scale (NRS) and Patient Global Impression of Change (PGIC) scores at four time points. The ability to perform a 3-meter walk test and the use of analgesics were also evaluated. Group comparison and repeated-measure analysis were carried out using nonparametric statistics.

**Results:**

The active TENS group exhibited significantly higher PGIC scores after 30 minutes, which persisted throughout the intervention (all *p* ≤ 0.001). A reduction in NRS appeared after one hour of active TENS, persisting throughout the intervention (all *p* ≤ 0.05). The median group differences in pain ratings were greater than the minimum clinically important difference, and the analysis of pain trajectories confirmed clinical significance at the individual level. Moreover, patients in the active TENS group were more likely able to perform a 3-meter walk test by the end of the intervention (*p* = 0.04). Analysis of the opioid-sparing effect of TENS was inconclusive (*p* = 0.066). No postoperative surgical complications or TENS-related side effects were observed during the study.

**Conclusion:**

Mixed-frequency TENS integrated in pants could potentially be an interesting addition to the arsenal of treatments for multimodal analgesia following hip surgery. This trial is registered with NCT05678101.

## 1. Introduction

Hip fracture is most often addressed surgically [1, 2] and is commonly associated with a short-term increase in mortality and long-term reduction in health-related quality of life [3–6]. According to the Swedish National Hip Fracture Registry [2], 5.8% of the patients registered in 2019 were experiencing severe pain either constantly or during activity at the 4-month follow-up; 42% were experiencing tolerable pain during activity, and 35% were pain-free.

Besides hip fracture, other patients undergoing hip surgery are also at risk of developing chronic postoperative pain (CPOP), with an estimated incidence of 5%–85%, varying with populations and types of surgeries [7–9]. Orthopedic surgery and severe pain on postoperative day 1 are important risk factors of CPOP [10]. A 10% increase in the amount of time spent in severe pain on postoperative day 1 has been associated with a 30% increase in the incidence of CPOP at a 12-month follow-up. Yet, a 2021 review on the prevention of CPOP [11] reported that there were no pharmaceutical pain treatments that could be universally or specifically recommended to reduce the incidence of CPOP.

To effectively manage acute and chronic pain disorders, transcutaneous electrical nerve stimulation (TENS) has been viewed a safe nonpharmacological addition to standard care [12–14]. Conventional TENS with high frequency (50−100 Hz) activates non-noxious afferents considered to inhibit nociceptive transmission at the spinal cord level, i.e., peripheral gate-control. Mixed-frequency TENS adds a low-frequency (mostly 2−10 Hz) stimulation that activates small-diameter, high-threshold peripheral afferents considered to trigger brainstem opioid descending pain inhibitory pathways, i.e., central gate-control. Studies have indicated that mixed-frequency TENS may be effective to mitigate postoperative pain [15, 16].

In 2019, a double-blinded, randomized trial assessed the effects of TENS on acute postoperative pain intensity and mobility after hip fracture [17]. A statistically significant pain reduction was observed during ambulation, with a 30-minute high-frequency TENS stimulation daily on the first 5 postoperative days. However, whether TENS on the first postoperative days (POD 0–2) can yield improved analgesia during both rest and activity is still unclear.

The primary aim of the present study was to assess pain reduction and patient global impression of change following mixed-frequency TENS treatment (or placebo TENS) at rest and during activity on the first postoperative days following hip surgery. A secondary aim was to assess improvement in mobility and to test the opioid-sparing effect associated with TENS treatment. Moreover, to simplify TENS application, empower elderly patients, and improve compliance during mobilization, the authors chose to administer TENS using wearable TENS-pants with modular textile electrodes. We hypothesized that adjunctive mixed-frequency TENS, using TENS-pants, would significantly enhance postoperative pain mitigation and improve mobilization after hip surgery.

## 2. Methods

### 2.1. Study Design and Setting

**Figure 1:**
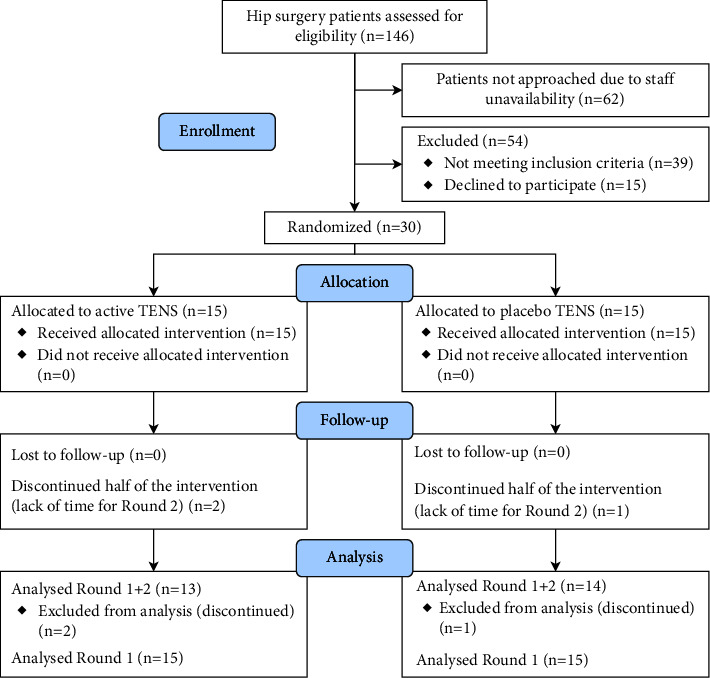
Flowchart of enrollment, allocation, follow-up, and analysis (following the CONSORT guidelines).

This study is a prospective, randomized, single-blinded, placebo-controlled, repeated-measures study, including 30 patients (see CONSORT [18] flowchart in Figure 1). The study design and measured outcomes were approved by the Swedish Ethical Review Authority (Dnr 2022-04307-01) and preregistered in ClinicalTrials.gov under the identifier NCT05678101. The study was conducted in an orthopedic department at a major university hospital in Stockholm, Sweden. The data analysis was not preregistered.

Recruitment and data collection were carried out between October 2022 and February 2023 by a research nurse. Patients aged over 18 years, scheduled for surgery following acute hip fracture, or scheduled for elective total hip arthroplasty or revision hip replacement, were eligible for study inclusion. Exclusion criteria were terminal illness, pacemaker, intracardiac defibrillator, class 3 or 4 heart failure, postoperative delirium syndrome, cognitive impairment, epidural catheter, documented drug/narcotic abuse, pregnancy, open skin wounds around incision area after surgery, or inability to understand Swedish.

On the first postoperative days (POD 0–2, see Table 1), eligible patients received oral and written information about the study and how to participate, including information on data collection and personal data handling in accordance with the EU's General Data Protection Regulation (GDPR). Each patient choosing to participate in the study signed an informed consent. Study participation was voluntary, and patients could cancel their participation at any time without specifying a reason.

### 2.2. Treatments

#### 2.2.1. Active TENS

Mixed-frequency TENS was used with the aim of triggering both peripheral gate-control of the relevant osteotomes, myotomes, and dermatomes (L2–L4) [19, 20], as well as descending pain inhibitory pathways. The active mixed-frequency TENS produced mixed bursts of 80 Hz and 2 Hz frequencies, with individually set intensities, that alternated every three seconds, using a Chattanooga Physio® electro-stimulation device—Chattanooga, DJO Nordic, Malmö, Sweden [21].

The intensity of the 80 Hz stimulation was set first by gradually increasing the intensity until the patients reported a tingling sensation; then the procedure was repeated for the 2 Hz stimulation. The TENS intensity was adjusted to each patient's highest tolerable stimulation level. TENS stimulation intensity was chosen strong enough to be felt but remained at a level that was not perceived as painful nor uncomfortable.

#### 2.2.2. Placebo TENS

For placebo TENS, the electro-stimulation device was kept powered on but not programmed to perform any stimulation. More specifically, patients in the placebo group were told that the TENS-pants were a new experimental device designed to make TENS stimulation almost imperceptible, but that they might still experience some light sensations such as itch or tingling. Beyond the difference in TENS treatment, all patients in the study followed the same protocol, including putting on the TENS-pants and performing standardized walk tests.

#### 2.2.3. TENS-Pants

To facilitate walking during electrostimulation and to reduce the impression of solemnity for elderly patients, the TENS treatment was applied with designed pants (referred to as TENS-pants) using textile electrodes. Four pairs of pants (2 of size S/M and 2 of size L/XL) with modular textile electrodes were custom-made for this study. The two sizes were created based on a size chart for garment construction [22]. Size S/M could fit women and men with a hip measurement range of 92−108 cm. Size L/XL could fit women and men with a hip measurement range of 108−116 cm. The pants were designed with an opening along the side of the legs, from waist to knee level, similarly to diapers, to be easily put on by bedridden patients with the help of a caregiver. The back part of the pants overlapped the front part and was closed by sticking tabs of mushroom-shaped hook tape to the fabric on the front. In this way, the garment pressure could be adjusted by tightening and loosening the tabs of hook tape to optimize contact between the electrodes and the skin.

The lining of the TENS-pants, most adjacent to the hip areas, was made from a brushed pile warp knit to enable the free placement of modular textile electrodes that could attach to the warp knit with hook tape. Each pair of TENS-pants included two pairs of 8 × 12 × 1 cm textile electrodes. Two electrodes were placed ventral and two dorsal, relative to the surgical incision, approximately 5 cm from the surgical dressing [23].

**Figure 2:**
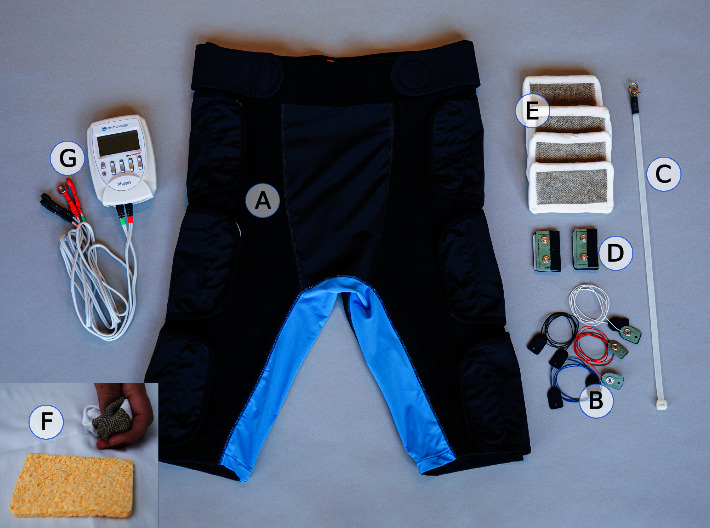
Pants designed for this study, with all necessary components. (A) Pants designed for dressing the patient in bed, making use of two rows of four straps each, placed along the side of the legs, also allowing fine adjustment of fit (supporting a good electrode contact). (B) Inner wires with two symmetrical ends with snap buttons for connecting electrodes with the electrostimulation device. (C) Sliding tool used to insert the inner wires in dedicated channels in the lining of the double layer of the pants. (D) Edge connectors (two) to be placed at the lower hemline, interfacing the inner wires with the electrostimulation device. A distal position keeps most cables and rigid parts away from the surgical wound. (E) Textile electrodes (four) with the knitted conductive side upward (brownish color). (F) Textile electrodes are wrapped around sponges for moisture storage and have the advantage of being soft and very flexible. (G) Electrostimulation device with outer wires and ends compatible with the snap buttons of the edge connectors. The electrostimulation device and outer wires are held by a nurse during the walk tests.

The electrodes were made of a single knitted fabric—conductive area: Shieldex® [24] 117/17dtex, 2-ply, z-twisted; surrounding material: polyamide—enveloping a cellulose sponge with an open cell structure. Electrodes were wetted [25] with 20 mL of a 0.9% NaCl solution before application on the patients' skin. Each of the electrode pairs were connected to one of the four available channels of the electro-stimulation device. A picture of the TENS-pants, electrodes, and other pieces of equipment used in the present study can be found in Figure 2.

**Figure 3:**
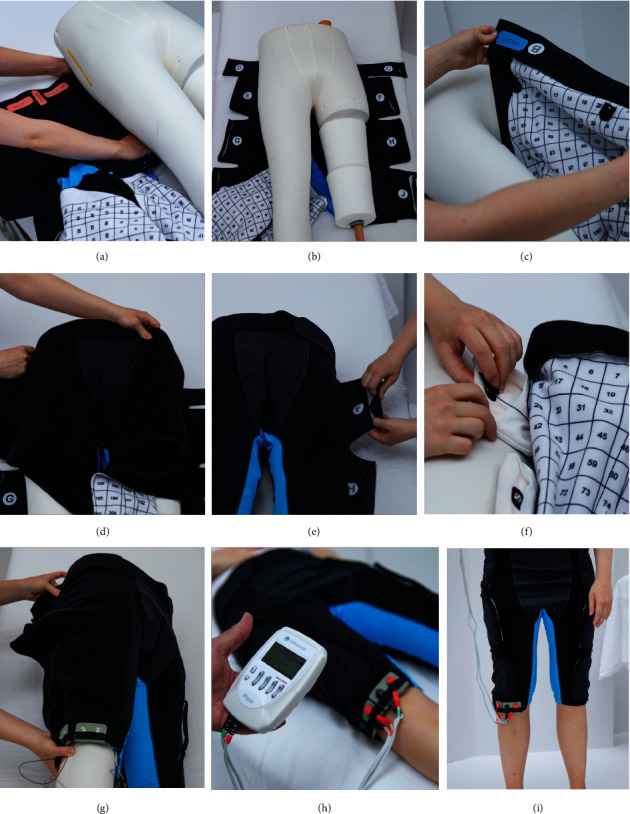
Series of pictures demonstrating step-by-step (from (a–i)) how the TENS-pants can be fitted on a patient lying down on the bed. The pants can be opened on the lateral side of the legs and laid flat under the patient (a, b). Alternatively, the TENS-pants can be positioned with the patient either standing or rolling to their side. With the back of the pants in place, the front side is pulled up between the patient's legs (c, d). The belt and the side of the pants not requiring electrodes are fastened first (e), and 4 textile electrodes are positioned around the surgical wound in a TENS setup (f) [23]. The side of the pants covering the electrodes is fastened as well (g), and the stimulation device is connected to the edge connectors and operated by the nurse (h). The TENS-pants are designed both for in-bed stimulation and stimulation during activity (i).

Depending on the patient's mobility, the TENS-pants were fitted on the patient in one of three different manners. If the patient was able to get out of bed and stand for a short time, the pants were fitted around the patient's waist and closed around the legs while the patient was standing. The electrodes were either placed at the same time or later when the patient was at rest lying on the bed again. If the patient felt uncomfortable with standing but was able to lie on their side, the pants were folded and pulled between the patient's legs to facilitate fitting with minimum pressure on the side of the operated hip. In case the patient was unable to lie on their side, the pants were laid flat on the bed, similarly to a diaper, and pushed under the patient with the help of a second nurse (Figure 3).

### 2.3. Outcome Measures

**Figure 4:**
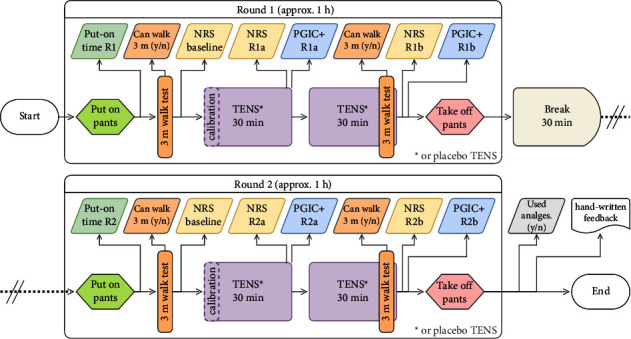
Flowchart illustrating the timeline of an entire intervention.

Self-reported pain rating on a pain Numerical Rating Scale (NRS) and Patient Global Impression of Change (PGIC) were the primary endpoints [26] of the present study. Mobility, measured in terms of the ability to perform a standardized 3-meter walk test [27], as well as the opioid-sparing effect [28] associated with TENS treatment (i.e., use of pharmaceutical analgesics over the entire length of the intervention) constituted secondary endpoints. The chronology of the intervention and measurements is illustrated in Figure 4.

#### 2.3.1. Pain NRS

Pain intensity assessments were carried out using an 11-item NRS. Patients were instructed to rate the intensity of their pain between 0 (no pain) and 10 (worst imaginable pain) [29, 30].

#### 2.3.2. PGIC Scale

Several versions of a 7-point Patient Global Impression of Change (PGIC or PGI-C) can be found in medical and pain-related literature. What could be referred to as a “standard” PGIC scale is classically centered around “no change” and uses three items for assessing improvement and three items for worsening. Items are usually ordered from 1 being “very much better” to 7 being “very much worse” [31], but the same scale can also be found in reverse order, i.e., with lower score representing worsening [32], and sometimes numbered from −3 to +3 to reflect the symmetry around “no change.” Conversely, the PGIC scale used in the present study is a nonsymmetrical scale proposed by Hurst and Bolton [33] to allow for more nuances of patient-reported impressions. The scale ranges from 1: “No change (or condition has gotten worse)” to 7: “A great deal better, and a considerable improvement that has made all the difference.” To avoid confusion with other PGIC scales, and with regard to its focus on positive improvement, the scale used in the present study will be referred to as “PGIC+.”

### 2.4. Study Procedure

Following inclusion and informed consent, patients were randomly allocated to either the intervention group or the placebo group in a 1 : 1 ratio, through the draw of secret envelopes prepared by a third person. Patients were kept unaware of their allocated group throughout the study. The study procedure consisted of two “rounds,” each round containing two 30-minute sessions of either active TENS (treatment group) or placebo TENS (placebo group), in addition to regular postoperative standard of care.

The study procedure began by applying the TENS-pants to the patients, when they felt ready, with assistance from the research nurse conducting the study. After the pants were fitted, round 1 started by offering the patients to conduct a standardized 3-meter walk test (WALK 3 m) [27], directly followed by a baseline pain assessment using a NRS. If a patient was unable to perform the walk test, baseline pain was assessed at rest instead.

Afterwards, with the patients resting in a prone position in their hospital bed, the first 30-minute session (“Round 1 Session a,” abbreviated as “R1a”) of active/placebo TENS began. A second NRS pain assessment was carried out within the last five minutes of the first TENS session, directly followed by an assessment of the impression of change relative to the perceived pain prior to the fitting of the TENS-pants on a PGIC + scale [33]. After completion of the PGIC + inquiry, a second 30-minute session (“R1b”) started. Within the last five minutes of this second session, patients were again asked to perform the 3-meter walk test, and after completion, in a sitting position, to rate again their pain intensity (NRS) and impression of change relative to the perceived pain prior to the fitting of the TENS-pants (PGIC+). This assessment marked the end of round 1.

After round 1, patients were helped to take off the pants and had a 30-minute break, whereafter a second round (“Round 2,” abbreviated as “R2”) was conducted following the exact same procedure as round 1. All supplementary analgesics consumed during the 2 hours of intervention (rounds 1 and 2) were documented to aid in evaluating the opioid-sparing effect of the intervention.

### 2.5. Statistical Analysis and Graphical Representation

#### 2.5.1. Software and Tools

The largest part of the statistical analysis was carried out using Python version 3.10.9. Nonparametric statistical test results were obtained from SciPy version 1.10.0 [34]. ANOVA testing was performed with Statsmodels version 0.13.2 [35]. Data handling, descriptive statistics, and necessary calculations were performed with Pandas v1.5.3 [36] and NumPy v1.24.1 [37]. Graphs were generated with seaborn v0.12.2 [38], matplotlib v3.6.3 [39], pyCirclize v0.3.1 for the chord diagrams, and a slightly modified version of psankey v1.0.0 for the Sankey diagrams.

#### 2.5.2. Sample Size Calculation

Based on earlier studies on TENS, and our own pilot study, we estimated a 30% reduction in pain ratings during activity [17, 40]. Thus, twelve patients per group would be required to detect an absolute difference of 2.1 in NRS scores, which would be clinically meaningful—two‐sided type‐I error rate=5%; power=80% [31, 41–44]. We added 20% additional patients, resulting in a total of 30 patients recruited.

#### 2.5.3. On the Choice of Appropriate Statistical Tests and Indicators

A certain controversy exists in the literature about the analysis of NRS pain ratings [45–48]. The present analysis was based on the assumption [49] that self-reported pain ratings should be considered ordinal measurements [45, 48]. The same reasoning was applied to PGIC + results. For this reason, NRS and PGIC + results were analyzed using nonparametric indicators: median, interquartile range (IQR), and nonparametric tests: Mood's test for the equality of medians, the Wilcoxon–Mann–Whitney test for stochastic equality, and Page's test for rank-order trends [50–52].

Gender distribution in the placebo group and treatment group was analyzed using Fisher's exact test [53]. Age distribution in the two groups was checked for normality using a Shapiro–Wilk test (*p*=0.003). A type II ANOVA was performed after rank-transformation [54]—Shapiro–Wilk after rank-transformation: *p*=0.235. Intercorrelation between variables was tested using Spearman's correlation coefficient, due to its usability and performance with categorical variables [55]. A robust version of Levene's test for the equality of variances [56] was also used in Table 1 in order to assess possible differences of repartition between the two groups in terms of the timing of the intervention.

When relevant, effect size was indicated using proportional odds ratios [57], Wilcoxon–Mann–Whitney odds abbreviated as WMW odds [58, 59], and number needed to treat (NNT) for compatibility with future meta-analyses [58, 60].

#### 2.5.4. WMW Odds, NNT, and Confidence Intervals

Confidence intervals for the WMW Odds were calculated based on the DeLong estimate of variance [61] for the WMW statistic (equation (1)):

(1) $$\begin{array}{c} \overset{\wedge }{Var_{DL}}\left(\overset{\wedge }{\theta }\right)=\frac{1}{m\left(m-1\right)}\overset{m}{\underset{i=1}{\sum }}{\left(\bar{V_{i.}}-\overset{\wedge }{\theta }\right)}^{2}+\frac{1}{n\left(n-1\right)}\overset{n}{\underset{j=1}{\sum }}{\left(\bar{V_{.j}}-\overset{\wedge }{\theta }\right)}^{2}\\ \text{With}:\\ \overset{\wedge }{\theta }=\frac{U}{\text{mn}}=\frac{1}{\text{mn}}\overset{m}{\underset{i=1}{\sum }}\overset{n}{\underset{j=1}{\sum }}V_{\text{ij}}\text{ and }V_{\text{ij}}=\left\{\begin{array}{cc} 1 & \text{ if }{\;X}_{i}<Y_{j},\\ 0.5 & \text{ if }X_{i}=Y_{j},\\ 0 & \text{ if }X_{i}>Y_{j}. \end{array}\right. \end{array}$$

where *U* is the Wilcoxon–Mann–Whitney statistic and *m*, *n* are the respective sizes of group *X* and group *Y*. $\overset{¯}{V_{i.}}$ and $\overset{¯}{V_{.j}}$ are shorthands for (1/*n*)∑_j_V_ij_ and (1/*m*)∑_i_V_ij_ , respectively. A confidence interval around the $\overset{\wedge }{\theta }$ estimate was then formed using a logit scale method [62] (equation (2)):

(2) $$\begin{array}{c} \theta \in \left[\frac{e^{\overset{\wedge }{\theta_{l}}}}{1+e^{\overset{\wedge }{\theta_{l}}}};\frac{e^{\overset{\wedge }{\theta_{u}}}}{1+e^{\overset{\wedge }{\theta_{u}}}}\right]\\ \text{With}:\\ \overset{\wedge }{\theta_{l/u}}=\ln\left(\frac{\overset{\wedge }{\theta }}{1-\overset{\wedge }{\theta }}\right)\pm z_{1-\alpha /2}\frac{\sqrt{\overset{\wedge }{\text{Va}r_{\text{DL}}}\left(\overset{\wedge }{\theta }\right)}}{\overset{\wedge }{\theta }\left(1-\overset{\wedge }{\theta }\right)}. \end{array}$$

The boundaries of the confidence interval for *θ* were then substituted in (3) to determine a confidence interval for the WMW odds:

(3) $$\begin{array}{c} \text{WMW Odds}=\frac{\theta }{1-\theta }. \end{array}$$

The number needed to treat (or number needed to harm, NNH) and its confidence interval were calculated from the WMW odds [58] (equation (4)):

(4) $$\begin{array}{c} \text{NNT}=1+\frac{2}{\text{WMW Odds}-1}. \end{array}$$

#### 2.5.5. Line Plots with Cumulated Frequencies (Time Series)

The median NRS (or PGIC+) scores of both groups were plotted against time. In these plots, results from the active TENS group are presented in green and results from the placebo group in orange. A dotted line is used (by opposition to a plain line) to indicate the interval between the last measurement of round 1 and the first measurement of round 2. A shaded area around both median lines indicates the interquartile range (IQR) for the scores of their respective group. The top edge of the graph presents the exact timeline of TENS sessions. The right edge of the graph presents the cumulated frequency of each possible score on the NRS (or PGIC+) scale.

#### 2.5.6. Circular Correlation Maps (Chord Diagrams)

Spearman's rank-order correlations between most of the variables and outcomes of interest for round 1 and round 2 were calculated in Python (SciPy) and mapped using chord diagrams. In these diagrams, the width of a link is directly proportional to the amplitude of the correlation (value between 0 and 1). The rectangles at the end of each link indicate the two-sided significance level (number of rectangles) and the sign (filling of the rectangles) of the correlation between two variables. Binary categorical variables, such as “Placebo/Active” or “Gender (M/F),” were encoded with 0 and 1 in the same order as described (Placebo=0, Active=1; M=0, F=1, etc.). “Walk” variables refer to the patient's ability to perform the 3-meter walk test (not able=0, able=1).

#### 2.5.7. Circular Map of Statistical Outcome Difference (Chord Diagrams)

Variables of interest were used to split the data (value of binary variable, or median split otherwise) and the resulting groups were compared with a Wilcoxon–Mann–Whitney *U*-test in Python (SciPy). The effect sizes associated with each comparison were mapped using chord diagrams. In these diagrams, the width of each end of a link is proportional to the log of the Wilcoxon–Mann–Whitney odds, where the variable at the other end of the link is used to split the data (hence, the width of each end indicates how significantly one variable is affected by a split based on another variable). The rectangles at the end of each link indicate the two-sided significance level (number of rectangles) and the direction of the difference (i.e., outcome *B* | *A*^+^ greater or lower than outcome *B* | *A*^−^). Binary categorical variables, such as “Placebo/Active” or “Analgesic (No/Yes),” were encoded with 0 and 1 in the same order as described (Placebo=0, Active=1, etc.). “Walk” variables refer to the patient's ability to perform the 3-meter walk test (*not* *able*=0, *able*=1).

#### 2.5.8. Sankey Diagrams

NRS pain ratings were split into four categories of pain severity (0: “no pain,” 1–3: “mild pain,” 4–6: “moderate pain,” 7–10: “severe pain”) 42, and Sankey diagrams were generated using these categories to visualize the pain trajectory 63 of patients in the active TENS group and placebo TENS group. In these diagrams, categories of pain severity are aligned vertically for better readability. Page's test for trends was used on the same categories (“no pain,” “mild pain,” “moderate pain,” and “severe pain”) to test the hypothesis that patients are globally transitioning to lower pain categories over time, relative to their own baseline.

#### 2.5.9. Notations

When reporting effect size, 95% confidence intervals are given between square brackets after the corresponding point estimate. Interquartile ranges are given in round brackets after a median. The notation “+inf” might also be encountered in the reporting of the odds ratio (OR), number needed to treat (NNT), or number needed to harm (NNH) and is a shorthand for “positive infinity.” In Table 2, the notation NNT/NNH=x(a], [*b*) indicates that the NNT (if positive) or NNH (if negative) estimate is *x* and that the corresponding confidence intervals are [*a*; +inf] for the NNH and [*b*; +inf] for the NNT.

## 3. Results

### 3.1. Patients

A total of 146 patients were assessed for eligibility for the trial, and 54 patients were excluded for various reasons. Thirty participants were enrolled in the trial with 15 patients in each group. The intervention was carried out as designed. No participants were lost during the postoperative testing round 1, but three participants discontinued due to time restraints after round 1 (Figure 1: CONSORT flowchart, Figure 4). Most patients received regional anesthesia using neuraxial block and a combined epidural-spinal technique during surgery. Characteristics of the patients allocated to the Active TENS and Placebo TENS groups are presented in Table 1. No postoperative surgical complications or TENS-related side effects were observed during the study.

### 3.2. Numerical Rating Scale (NRS) Scores at Rest and after Activity

**Figure 5:**
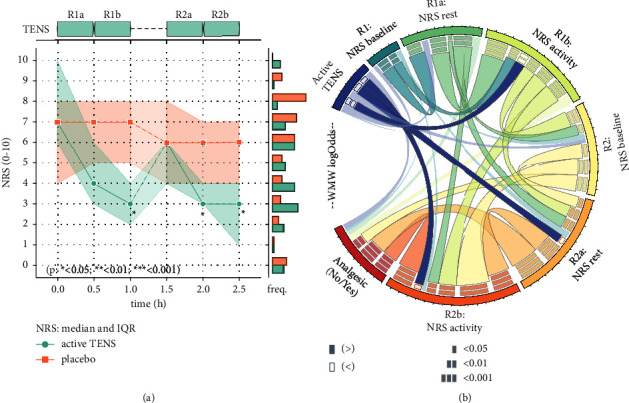
(a) Evolution of median pain ratings over time on a Numerical Rating Scale (NRS). (b) Statistically significant increase (“>”) or decrease (“<”) in pain ratings (NRS) and analgesic use, according to a two-sided Mann–Whitney test. Links related to “Active TENS” indicate significant differences between the Active TENS group and the Placebo TENS group. The use of active TENS appears to be associated with a sharp and significant reduction in pain ratings (NRS) after the first hour of electrostimulation (R1b, R2a, R2b). Use of analgesics appears strongly correlated with the pain ratings during round 2. More details about the construction and reading of each graph in the Methods section.

The baseline pain intensity levels before testing round 1 were similar between the two groups (see Table 1) with a median NRS [IQR] | mean (std) of 7 [6–10] | 7.33 (1.99) in the Active TENS group and of 7 [4–8] | 5.73 (3.01) in the Placebo TENS group at time=0 h (Figure 5(a)). After 30 minutes of TENS, the NRS at rest was not statistically significantly lower in the Active TENS group: 4 [3–6] | 4.73 (2.49), as compared to the Placebo TENS group: 7 [5–8] | 6.27 (2.60) (time=0.5 h, Mood's test for the equality of medians *p*=0.067). After 60 minutes of TENS, the NRS after activity was however significantly different between the two groups, 3 [2–4] | 3.67 (1.80) compared to 7 [5–8] | 6.13 (2.83) in the Active vs. Placebo TENS groups (time=1.0 h, *p*=0.029).

The baseline pain intensity levels before testing round 2 were comparable between the two groups with median NRS [IQR] | mean (std) of 6 [4–6] | 5.08 (1.44) in the Active TENS group and of 6 [4–8] | 5.43 (2.56) in the Placebo TENS group (time=1.5 h, Figure 5(a)). After 30 minutes of TENS, the NRS at rest was significantly different between the groups, 3 [3-4] | 3.23 (2.31) in the Active TENS group and 6 [4–7] | 5.07 (2.70) in the Placebo TENS group (time=2.0 h, *p*=0.011). After 60 minutes of TENS, the NRS after activity was still significantly different between the groups, 3 [1–4] | 2.92 (2.29) in the Active TENS group compared to 6 [4–7] | 5.00 (2.69) in the Placebo TENS group (time=2.5 h, *p*=0.033). Respective values and confidence intervals for the effect size can be found in Table 2.

**Figure 6:**
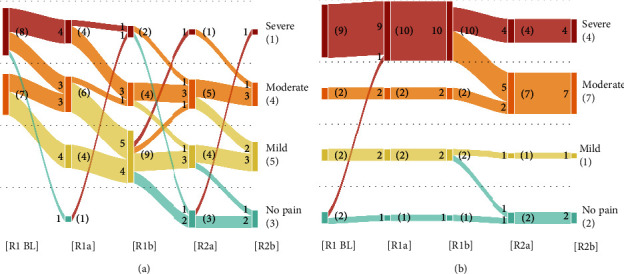
(a) Individual pain trajectories in the Active TENS group. (b) Individual pain trajectories in the Placebo group. The numbers on each diagram represent the number of patients with a given self-reported pain severity (estimated from NRS), as well as their transitions between different steps of the intervention. Patients in the active TENS group quickly transitioned from “severe pain” to “moderate” or “mild pain.” Most pain levels remained consistent between R1b and R2a despite the 30-minute break. More details about the construction and reading of these Sankey diagrams in the Methods section.

Overall, pain ratings appeared to start dropping as soon as TENS was applied in the Active TENS group. A statistically significant difference was achieved after two 30-minute TENS sessions and can be observed both through the difference in median NRS scores and in a Wilcoxon–Mann–Whitney test (Figures 5(a) and 5(b)). Effect size calculation showed a consistently stronger effect size when TENS was used both at rest and during activity, compared to TENS at rest alone (Figure 5(b), Table 2). Nonetheless, all differences in median pain ratings during TENS (time=0.5 h, t=1 h, time=2 h, and time=2.5 h) were greater than the minimum clinically important difference (MCID, estimates varying around 1.4 to 2.1 for the NRS) found in the literature [31, 41–44]. A slow natural decrease in pain ratings also appeared over time in both groups (observable in Figures 5(a), 6(a), and 6(b)), but the difference between the Active TENS group and the Placebo group remained significant.

### 3.3. Pain Trajectories

The pain trajectories of the patients strengthened the observations made by depicting changes in pain over time in the Active TENS and Placebo TENS groups (Figure 6). Patients in the Active TENS group all reported moderate (*n*=7) to severe (*n*=8) pain levels at the beginning of the intervention. After the first 30-minute TENS session, approximately half of the Active TENS group (8/15) had transitioned to lower pain levels (severe pain to moderate or no pain; moderate pain to mild pain). At the end of the 2.5-hour intervention, more than half of the patients in the Active TENS group (8/13) reported mild pain or less. The two patients who could not participate in round 2 both reported mild pain at the end of round 1. One patient was seen oscillating between “severe pain” and “no pain,” possibly due to higher pain levels during activity (walk tests).

By contrast, pain trajectories in the Placebo TENS group appeared remarkably monotonous. Patients with moderate (*n*=2) or severe pain (*n*=9) at the beginning of the intervention all remained at moderate and severe pain levels, respectively, during the first round. The same patients reported again moderate (*n*=7) or severe pain (*n*=4) at the end of the intervention. Approximately half of the patients reporting severe pain at the end of round 1 (*n*=10) transitioned to moderate pain levels (5/9) by the first half of round 2. The patient who could not participate in round 2 reported severe pain at the end of round 1.

An analysis of the individual pain trajectories for round 1 and round 2 with the Page test (same four categories, tested alternative hypothesis: decreasing rank-order over time, i.e., baseline ≥ session *a* ≥ session *b*, with at least one strict inequality) indicated a statistically significant decrease in pain categories for both round 1 (*p* < 0.001) and round 2 (*p*=0.019) in the Active TENS group (see Table 2). In the Placebo group, there was no statistical evidence for such a trend in neither round 1 (*p*=0.674) nor round 2 (*p*=0.320).

Pain trajectories also seemed to indicate a relative persistence of the effects of TENS between round 1 and round 2. Most notably, five of the seven patients who reached “mild” pain levels by the end of round 1 in the Active TENS group (i.e., supposedly as a result of TENS treatment) and continued into round 2 reported “mild” pain again, or no pain at all, after the resumption of TENS treatment.

### 3.4. Patients' Global Impression of Change

**Figure 7:**
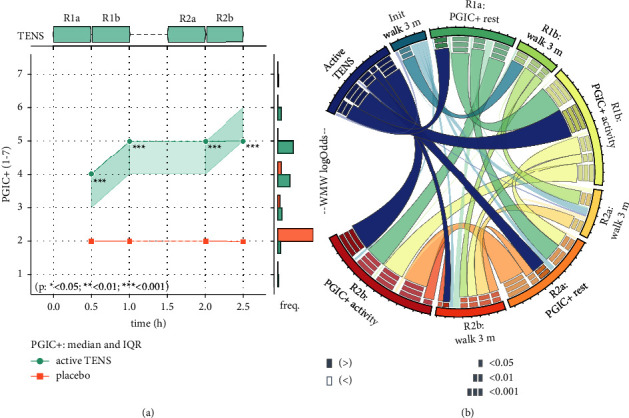
(a) Evolution of median patient global impression of change (PGIC+) over time. (b) Statistically significant increase (“>”) or decrease (“<”) in PGIC+ and ability to perform a 3-meter walk test, according to a two-sided Mann–Whitney test. The use of active TENS appears to be associated with a significant increase in PGIC + at all time points after the first half-an-hour of electrostimulation and is also associated with a significant improvement in the ability to perform the 3-meter walk test after 2 TENS sessions of one hour each. More details about the construction and reading of each graph in the Methods section.

The evolution of the patients' global impression of change (PGIC+, 1–7) over the time of intervention corroborated an effect of the intervention as observed in the differences of NRS scores between the two groups (Figure 7(a)). After 30 minutes of TENS, the PGIC + at rest was significantly different between the groups with a median PGIC + [IQR] | mean (std) of 4 [3-4] | 3.60 (1.06) in the Active TENS group and of 2 [2-2] | 2.13 (0.52) in the Placebo TENS group (*time*=0.5h, Mood's test for the equality of medians *p* < 0.001). After 60 minutes of TENS, the PGIC + after activity demonstrated a greater difference between the groups with 5 [4-5] | 4.47 (0.64) in the Active TENS group and 2 [2-2] | 2.13 (0.52) in the Placebo TENS group (time=1.0 h, *p* < 0.001). After 30 minutes of TENS in round 2, the PGIC + at rest was still significantly different between the groups with 5 [4-5] | 4.15 (1.41) in the Active TENS group and 2 [2-2] | 2.29 (0.61) in the Placebo TENS group (time=2.0 h, *p*=0.001). After 60 minutes of TENS in round 2, PGIC + after activity demonstrated the same difference between the groups with 5 [5-6] | 5.15 (1.07) in the Active TENS and 2 [2-2] | 2.36 (0.74) in the Placebo TENS groups (time=2.5 h, *p* < 0.001).

At the end of the intervention, 10 patients out of 13 in the Active TENS group reported a PGIC+ of 5 (“Moderately better, and a slight but noticeable change”) or greater, 5 patients out of 13 reported a PGIC+ of 6 (“Better, and a definite improvement that has made a real and worthwhile difference”) or greater, and no patient reported any score below 3 (“A little better, but no noticeable change”). Statistical calculations verified the differences in PGIC + between the two groups and additionally provided effect sizes (see Figure 7(b), Table 2).

### 3.5. Ability to Perform the Standardized 3-Meter Walk Test

At the baseline before round 1, there were no significant differences in the patients' ability to perform the 3-meter walk test, with 4/15 patients able to perform the walk test in the Active TENS group and 6/15 in the Placebo group. After a total of 60 minutes of TENS, the 3-meter walk test did not demonstrate any significant difference between the groups. Similarly, at the baseline before round 2, there were no significant differences in the patients' ability to perform the 3-meter walk test. After 60 minutes of TENS in round 2, however, significantly more patients in the Active TENS group (8/13) compared to the Placebo TENS group (3/14) were able to perform the 3-meter walk test (*p*=0.040) (Table 2). The WMW odds estimate is 2.34, implying a two-fold increase in the relative probability of observing patients in the Active TENS group with a greater ability to perform the 3-meter walk test. The confidence interval for the NNT remains positive, indicating a low probability that TENS could have any detrimental effect on the ability to walk.

In the Active TENS group, the two patients that could not participate in the second round of the study had balanced results (i.e., one could perform both the initial walk test and the R1b walk test; the other patient could not). In the Placebo group, the patient that did not participate in the second round of the study could not perform the first two walk tests.

### 3.6. Use of Analgesics

When comparing the two groups over the entire duration of the intervention, the reduction in the use of analgesics was not statistically significant, with 8 patients out of 15 requesting analgesics within the duration of the study in the Placebo group, and 3 patients out of 15 in the Active TENS group (*p*=0.066). The results, nonetheless, showed a trend in favor of a reduction of the use of analgesics, as observed in the calculated effect size (Table 2).

### 3.7. Test of Preliminary Assumptions

#### 3.7.1. Representativity of the Population Sample

The gender and age distributions in the two groups (active TENS/placebo) did not show any significant deviation from what could be expected of a random sample of the target population. Furthermore, the age distribution was comparable with statistics obtained from the Swedish National Registry for Hip Fractures (average age of hip fracture patients in Sweden: 82 years, which is within one standard deviation from the sample average, see Table 1). This indicates that the observed results are unlikely to have been influenced by a selection bias or by an extreme case of “chance” in the randomization of the study participants.

#### 3.7.2. Relevance and Consistency of Outcome Variables

The high levels of intercorrelation between the outcome variables (pain measurement, impression of change, ability to perform a walk test, and use of analgesics; see Figures S1a and S1b) suggest that the primary and secondary outcomes of the present study could be seen as different aspects of the same “problem” and that the measured variables are relevant. Intercorrelation through time (multiple measurements of the same nature) also indicates consistency in the measurements (i.e., a patient with a high pain rating is more likely to have had a high pain rating in the previous measurement, and to have a high pain rating in the next measurement, rather than purely random ratings). This intercorrelation is also visible in Figures 5(b) and 7(b).

#### 3.7.3. Negligibility of Variables Nonrelated to Treatment

The low level of correlation between individual variables (age, gender, side of surgery) and outcome variables (pain ratings, impression of change, ability to walk, use of analgesics), especially in round 2 (Figure S1b), supports the assumption that group allocation (and therefore TENS treatment) was the primary source of differences in outcomes between the two groups. The few significant correlations that can be found during round 1 are not unknown to the literature—effect of age on pain ratings [63, 64] and ability to walk [5, 65].

## 4. Discussion

This prospective, randomized, single-blinded, placebo-controlled study demonstrated that mixed-frequency TENS integrated in pants, as an adjunct to standard care, is associated with a statistically significant improvement in both pain scores (NRS) and patient global impression of change (PGIC+), starting from one hour of TENS at rest and during activity, on the first postoperative days following hip surgery. Patients in the Active TENS group also showed an increased ability to perform a 3-meter walk by the end of the 2.5-hour intervention. Moreover, a trend was observed towards a possible opioid-sparing effect of TENS. These findings support the use of active TENS as part of a multimodal approach to postoperative pain management.

### 4.1. Pain Relief over Time

The observed tendency towards pain relief in the Active TENS group, statistically significant after 60 minutes of stimulation, is consistent with the conclusions of previous studies on the use of TENS for the treatment of acute pain [14, 17, 66, 67]. The study of Elboim-Gabyzon et al. [17] similarly demonstrated the pain reduction associated with TENS in hip fracture patients during the first 5 postoperative days but did not find evidence for a positive effect of TENS at rest. An important discrepancy between the two studies might lie in the duration of the intervention; the present study was being characterized by a longer TENS treatment (2 hours in total with a 30-minute break) as opposed to 30 minutes daily for the study of Elboim-Gabyzon et al.

The observation that the second pain baseline seemed comparable between the Active TENS group and the Placebo group may suggest that the effect of TENS quickly dissipates when TENS is not active. However, the patients had to take off and put on the TENS-pants between the rounds and perform a 3-meter walk test before the second NRS baseline measurement. These activities might have impacted the NRS ratings of the second baseline. Conversely, the analysis of pain trajectories highlights a relative consistency between the last pain ratings of round 1 and the first pain ratings after the resumption of the TENS treatment in round 2. This could suggest that some of the beneficial aspects of TENS are persistent after half an hour of treatment interruption. Some studies have suggested a cumulative effect of TENS during long-term use in chronic pain, neuropathic pain, and osteoarthritis patients [68–71]. The relative persistence observed here in individual pain trajectories could be an example of the same effect.

The observation of pain trajectories also supports the thesis of a stronger and immediate tendency to pain reduction in the Active TENS group. This tendency remains apparent when pain ratings are categorized as “mild,” “moderate,” or “severe” pain, thus highlighting the clinical significance of the observed results. Other studies have suggested an immediate effect of TENS [72, 73] but only in the case of chronic low back pain.

### 4.2. Positivity of Patient Experience

In parallel to the improvement in pain ratings, PGIC + scores denote a clear and consistent difference between the Active TENS group and the Placebo group, starting from the first 30-minute TENS session. PGIC + ratings in the Placebo group remain remarkably consistent around 2 (“Almost the same, hardly any change at all”), while ratings from the Active TENS group seem to express a “noticeable change” in most cases and seem to increase over time. Perception of change follows the same trend as pain ratings in the calculation of effect sizes, with a seemingly larger effect size for PGIC + ratings after TENS at rest and during activity, compared with TENS at rest alone. The confidence intervals for the number needed to treat indicate that only 1 to 1.34 patients need to undergo TENS treatment for at least one patient to show improvement in the PGIC + outcome. This signifies a near-certainty that TENS treatment results in a greater perception of change in virtually all treated patients, at least during activity.

Overall, PGIC + scores underline the patient-centered benefits of early TENS treatment. Although the use of PGIC or PGIC + scales is not rare in the pain literature, few studies involving TENS report PGIC results. A study conducted in 2016 reported improvement in PGIC scores with TENS for the treatment of chronic low back pain and chronic pain in lower extremities [74].

### 4.3. Faster Recovery and Opioid Sparing

While the ability to perform a 3-meter walk test is considered a secondary endpoint in the present study, it is interesting to notice that a statistically significant difference appears between the two groups at the end of the second round. This difference is further emphasized since there were initially more patients in the Active TENS group than in the Placebo group that were unable to perform the walk. Possible factors could include the use of mixed-frequency TENS, which elicits both peripheral and central gate control of pain transmission, and maybe also a reduction of local inflammation and inflammation-related hyperalgesia [17, 75, 76]. Two other studies have previously reported positive effects of TENS on the ability to perform short walks in hip fracture patients [17] and chronic pain patients [74].

The statistical power of the present study for the detection of an opioid-sparing effect is relatively limited with a detection limit of Δ*n*=6 in the number of patients using analgesics between the Active TENS group and the Placebo group. The threshold for significance was not met here, with an observed difference of Δ*n*=5, but the calculated odds seem to indicate that TENS might be beneficial (and most probably not harmful) for a reduction in the use of analgesics after hip surgery. This hypothesis is also in line with the observation of a significant pain reduction, a positive impression of change for the patients, and is strengthened by earlier studies suggesting an opioid-sparing effect of TENS [12, 16].

### 4.4. Limitations of the Present Study

The present study suffered from the same limitation as most studies pertaining to the effects of TENS treatment, with a modest sample size of 30 included patients. The present study also had design-related limitations. Most notably, the effect of wearing the TENS-pants remains untested. Moreover, the fact that patients were required to take off the pants and put them on again between the two TENS sessions does not allow any meaningful analysis of changes in pain scores during the break time. A subset of each group underwent elective arthroplasty instead of fracture-related surgery, but this factor was not analyzed due to the very limited size of these subsets. Lastly, the interaction effect between TENS treatment and the use of on-demand analgesics was not analyzed either, for the same reason of subset size limitation.

### 4.5. Perspectives for Further Studies

#### 4.5.1. Possible Risk Reduction for the Development of Chronic Pain?

Comparatively, patients reporting severe pain at the baseline in the Active TENS group spent less time with severe pain than their counterparts from the Placebo group. From the data, one could roughly estimate a 65% reduction in the time spent with severe pain in the Active TENS group compared with the Placebo group over the duration of the intervention. Considering previous studies that emphasized the probable link between the severity of acute pain on the first postoperative days and the incidence of CPOP [10, 77, 78], this observation raises hope that TENS might potentially contribute to reducing the incidence of CPOP in hip surgery patients.

#### 4.5.2. Use of e-Textiles: Human and Societal Perspectives

Patients from both the Active TENS group and the Placebo group gave positive feedback about the use of the TENS-pants, at the end of the intervention. Assuming that future studies were to corroborate the usefulness of TENS during activity, the integration of TENS into garments could be used to ease mobility during stimulation, empower the patients, and overall improve the patients' experience and autonomy in the first days or weeks following surgery.

## 5. Conclusion

The results of the present study indicate that mixed-frequency TENS is effective against acute pain on the first postoperative days, with statistically and clinically observable effects after the first hour of TENS stimulation. In light of the challenges related to the use of pharmaceutical treatments for the prevention of CPOP, TENS, potentially applied using textile electrodes in pants, could be an interesting addition to the arsenal of treatments for multimodal analgesia following hip surgery [79].

## Figures and Tables

**Table 1:** Characteristics of patients included in the study and statistical check for unbalance between the two groups (Active TENS and Placebo).

Characteristics	Active TENS group	Placebo TENS group	*p* value	Test type
*N*	15	15	—	—

*Gender*				
Male	9	4	0.139	#1
Female	6	11		

Age, years				
Mean ± SD	77.3 ± 11.1	76.6 ± 15.6	0.744	#2
Median [IQR]	79 [74, 84]	77 [71, 88]	1.000	#3
[Min, max]	[44, 90]	[43, 97]	0.917	#4

*Hip operated on*				
Left	8	7	1.000	#1
Right	7	8		

*Reason for hip surgery*				
Fracture	11	12	1.000	#1
Elective arthroplasty	4	3		

*Time after surgery*				
Same day	0	1	0.577	#4
1 day	15	12	0.072	#5
2 days	0	2		

*Initial pain (NRS 0-10)*				
Mean ± SD	7.33 ± 1.99	5.73 ± 3.01	0.081	#2
Median [IQR]	7 [6, 10]	7 [4, 8]	1.000	#3
[Min, max]	[5, 10]	[0, 9]	0.286	#4

*Initial walk test*				
Able	4	6	0.700	#1
Not able	11	9		

Results do not seem to indicate any statistical unbalance between the two randomized groups with regard to the variables of interest. Statistical tests: #1: Fisher's exact test (independence). #2: ANOVA on ranks (equality of means, taking into account gender and age). #3: Mood's median test (equality of medians). #4: Wilcoxon–Mann–Whitney *U*-test (rank-order stochastic equality). #5: Median-based version of Levene's test (equality of variances).

**Table 2:** Main results and calculated effect size for comparisons in pain ratings (NRS), impression of change (PGIC+), pain trajectory (repeated measure on a 4-point scale from “no pain” to “severe pain”), ability to perform the 3 m walk-test, and use of analgesics between the Active TENS group and the Placebo group.

Data	WMW odds [95% CI]	OR [95% CI]	NNT(+)/NNH(−) [95% CI]	*p* value
*NRS (median)*				
R1a, time = 0.5 h	0.43 [0.23, 0.94]	0.18 [0.04, 0.87]	2.49 [1.59, 35.1]	0.067
R1b, time = 1.0 h	0.36 [0.20, 0.84]	0.13 [0.03, 0.67]	2.14 [1.50, 11.6]	^*∗*^0.028
R2a, time = 2.0 h	0.28 [0.12, 0.78]	0.07 [0.01, 0.49]	1.76 [1.28, 8.03]	^*∗*^0.011
R2b, time = 2.5 h	0.35 [0.17, 0.86]	0.12 [0.02, 0.68]	2.07 [1.42, 13.3]	^*∗*^0.033

*NRS (WMW)*				
R1a, time = 0.5 h	0.46 [0.18, 1.18]	—	2.71 (−12.4], [1.44)	0.085
R1b, time = 1.0 h	0.28 [0.10, 0.84]	—	1.79 [1.21, 11.1]	^*∗∗*^0.009
R2a, time = 2.0 h	0.37 [0.13, 1.07]	—	2.19 (−31.6], [1.30)	^*∗*^0.044
R2b, time = 2.5 h	0.36 [0.13, 0.99]	—	2.12 [1.30, 362]	^*∗*^0.038

*PGIC+ (median)*				
R1a, time = 0.5 h	6.89 [1.57, 19.8]	56.0 [5.13, 612]	1.34 [1.11, 4.50]	^*∗∗∗*^<0.001
R1b, time = 1.0 h	14.0 [2.21, 27.3]	196 [11.1, 3450]	1.15 [1.08, 2.66]	^*∗∗∗*^<0.001
R2a, time = 2.0 h	6.18 [1.47, 17.0]	43.3 [3.90, 482]	1.39 [1.13, 5.28]	^*∗∗∗*^<0.001
R2b, time = 2.5 h	—	inf.	—	^*∗∗∗*^<0.001

*PGIC+ (WMW)*				
R1a, time = 0.5 h	6.50 [2.22, 19.0]	—	1.36 [1.11, 2.64]	^*∗∗∗*^<0.001
R1b, time = 1.0 h	55.3 [6.84, 446]	—	1.04 [1.00, 1.34]	^*∗∗∗*^<0.001
R2a, time = 2.0 h	6.00 [1.50, 24.1]	—	1.40 [1.09, 5.03]	^*∗∗∗*^<0.001
R2b, time = 2.5 h	39.4 [7.17, 217]	—	1.05 [1.01, 1.32]	^*∗∗∗*^<0.001

*Pain trajectory*				
R1, TENS	2.05 [1.26, 3.63]	5.23 [1.97, 13.8]	2.90 [1.76, 8.59]	^*∗∗∗*^<0.001
R2, TENS	1.48 [0.99, 2.68]	2.80 [0.95, 8.24]	5.20 (−145], [2.19)	^*∗*^0.019
R1, placebo	0.98 [0.79, 1.62]	0.92 [0.25, 3.39]	−90.0 (−8.41], [4.22)	0.674
R2, placebo	1.10 [0.83, 1.90]	1.36 [0.41, 4.54]	21.0 (−11.0], [3.23)	0.320

*Ability to walk*				
R2b, time = 2.5 h	2.34 [1.01, 5.44]	5.87 [1.08, 32.0]	2.49 [1.45, 365]	^*∗*^0.040

*Use of analgesics*				
Entire study	0.50 [0.24, 1.06]	0.22 [0.04, 1.11]	3.00 (−33.1], [1.62)	0.066

*p* values:  ^*∗*^ < 0.05,  ^*∗*^ ^*∗*^ < 0.01,  ^*∗*^ ^*∗*^ ^*∗*^ < 0.001.

## Data Availability

The data presented in this paper contain sensitive information that fall under the European GDPR regulation and cannot be shared openly. The data that support the findings of this study are available from the corresponding author upon reasonable request.

## Abbreviations

BL: Baseline
CONSORT: Consolidated standards of reporting trials
CPOP: Chronic postoperative pain
MCID: Minimum clinically important difference
NNH: Number needed to harm
NNT: Number needed to treat
NRS: Numerical Rating Scale (here, for pain intensity)
OR: (Proportional) odds ratio
PGIC(+): Patient's global impression of change (+: with focus on positive changes)
POD: Postoperative day(s)
R1a, R2a, R2a, R2b: Round [1 or 2], session [a or b], see chronology
TENS: Transcutaneous electrical nerve stimulation
WMW: Wilcoxon–Mann–Whitney.

## Supplementary Materials

Supplementary Materials Figure S1: rank-order correlation (Spearman) between individual variables and outcomes measured during round 1 (a) and round 2 (b). The figure shows that pain ratings and impression of change are highly intercorrelated. Age, gender, and side of hip surgery have limited correlations with the outcome variables in round 1 and no significant correlation with the outcome variables in round 2.

## References

[1] Allahabadi S., Roostan M., Roddy E., Ward D. T., Rogers S., Kim C. (2022). Operative management of hip fractures within 24 hours in the elderly is achievable and associated with reduced opiate use. *Geriatric Orthopaedic Surgery & Rehabilitation*.

[2] Rikshöft (2020). *Annual Report Of the Swedish National Registry for Hip Fractures: Swedish National Registry for Hip Fractures RIKSHÖFT*.

[3] Alexiou K., Roushias A., Varitimidis S., Malizos K. (2018). Quality of life and psychological consequences in elderly patients after a hip fracture: a review. *Clinical Interventions in Aging*.

[4] Dyer S. M., Crotty M., Fairhall N. (2016). A critical review of the long-term disability outcomes following hip fracture. *BMC Geriatrics*.

[5] Gjertsen J.-E., Baste V., Fevang J. M., Furnes O., Engesæter L. B. (2016). Quality of life following hip fractures: results from the Norwegian hip fracture register. *BMC Musculoskeletal Disorders*.

[6] Griffin X. L., Parsons N., Achten J., Fernandez M., Costa M. L. (2015). Recovery of health-related quality of life in a United Kingdom hip fracture population. *The Bone & Joint Journal*.

[7] Clark J. D. (2021). Chronic postoperative pain: preventable or inevitable?. *Anesthesiology*.

[8] Gómez M., Izquierdo C. E., Mayoral Rojals V. (2022). Considerations for better management of postoperative pain in light of chronic postoperative pain: a narrative review. *Cureus*.

[9] Lopes A., Seligman Menezes M., Antonio Moreira de Barros G. (2021). Chronic postoperative pain: ubiquitous and scarcely appraised: narrative review. *Brazilian Journal of Anesthesiology (English Edition)*.

[10] Fletcher D., Stamer U. M., Pogatzki-Zahn E. (2015). Chronic postsurgical pain in Europe. *European Journal of Anaesthesiology*.

[11] Carley M. E., Chaparro L. E., Choinière M. (2021). Pharmacotherapy for the prevention of chronic pain after surgery in adults: an updated systematic review and meta-analysis. *Anesthesiology*.

[12] Jafra A., Ghai B., Bhatia N., Chanana N., Bansal D., Mehta V. (2022). Opioid sparing strategies for perioperative pain management other than regional anaesthesia: a narrative review. *Journal of Anaesthesiology Clinical Pharmacology*.

[13] Johnson M. I., Paley C. A., Jones G., Mulvey M. R., Wittkopf P. G. (2022). Efficacy and safety of transcutaneous electrical nerve stimulation (TENS) for acute and chronic pain in adults: a systematic review and meta-analysis of 381 studies (the meta-TENS study). *BMJ Open*.

[14] Vance C. G. T., Dailey D. L., Chimenti R. L., Van Gorp B. J., Crofford L. J., Sluka K. A. (2022). Using TENS for pain control: update on the state of the evidence. *Medicina*.

[15] Bjordal J. M., Johnson M. I., Ljunggreen A. E. (2003). Transcutaneous electrical nerve stimulation (TENS) can reduce postoperative analgesic consumption. A meta‐analysis with assessment of optimal treatment parameters for postoperative pain. *European Journal of Pain*.

[16] Hamza M. A., White P. F., Ahmed H. E., Ghoname E.-sA. (1999). Effect of the frequency of transcutaneous electrical nerve stimulation on the postoperative opioid analgesic requirement and recovery profile *anesthesiology*. *Anesthesiology*.

[17] Elboim-Gabyzon M., Andrawus Najjar S., Shtarker H. (2019). Effects of transcutaneous electrical nerve stimulation (TENS) on acute postoperative pain intensity and mobility after hip fracture: a double-blinded, randomized trial. *Clinical Interventions in Aging*.

[18] Cuschieri S. (2019). The CONSORT statement. *Saudi Journal of Anaesthesia*.

[19] Halaszynski T., Uskova A. (2016). *Regional Anesthesia for Hip Surgery*.

[20] Danzl M. M., Wiegand M. R. (2017). *Orthopedic Neurology*.

[21] (2023). Chattanooga. https://www.chattanoogarehab.com/physio-2535110-int.

[22] Öberg I., Ersman H., Cedervall M., Svensson C. (2010). *Mönster Och Konstruktioner För Damkläder*.

[23] Rakel B. A., Zimmerman B. M., Geasland K. (2014). Transcutaneous electrical nerve stimulation for the control of pain during rehabilitation after total knee arthroplasty: a randomized, blinded, placebo-controlled trial. *Pain*.

[24] (2023). Shieldex. supplier website. https://www.shieldex.de/en.

[25] Euler L., Juthberg R., Flodin J., Guo L., Ackermann P. W., Persson N.-K. (2021). Textile electrodes: influence of electrode construction and pressure on stimulation performance in neuromuscular electrical stimulation (NMES). *43rd Annual International Conference of the IEEE Engineering in Medicine & Biology Society*.

[26] Turk D. C., Dworkin R. H., McDermott M. P. (2008). Analyzing multiple endpoints in clinical trials of pain treatments: IMMPACT recommendations. *Pain*.

[27] Simpson J. M., Valentine J., Worsfold C. (2002). The Standardized Three-metre Walking Test for elderly people (WALK3m): repeatability and real change. *Clinical Rehabilitation*.

[28] Gewandter J. S., Smith S. M., Dworkin R. H. (2021). Research approaches for evaluating opioid sparing in clinical trials of acute and chronic pain treatments: initiative on Methods, Measurement, and Pain Assessment in Clinical Trials recommendations. *Pain*.

[29] Bendinger T., Plunkett N. (2016). Measurement in pain medicine. *BJA Education*.

[30] Ferreira-Valente M. A., Pais-Ribeiro J. L., Jensen M. P. (2011). Validity of four pain intensity rating scales. *Pain*.

[31] Farrar J. T., Young J. P., LaMoreaux L., Werth J. L., Poole M. R. (2001). Clinical importance of changes in chronic pain intensity measured on an 11-point numerical pain rating scale. *Pain*.

[32] Rampakakis E., Ste-Marie P. A., Sampalis J. S., Karellis A., Shir Y., Fitzcharles M.-A. (2015). Real-life assessment of the validity of patient global impression of change in fibromyalgia. *RMD Open*.

[33] Hurst H., Bolton J. (2004). Assessing the clinical significance of change scores recorded on subjective outcome measures. *Journal of Manipulative and Physiological Therapeutics*.

[34] Virtanen P., Gommers R., Oliphant T. E. (2020). SciPy 1.0: fundamental algorithms for scientific computing in Python. *Nature Methods*.

[35] Seabold S., Perktold J. Statsmodels: econometric and statistical modeling with Python. *Proceedings of the Python in Science Conference2010*.

[36] McKinney W. Data structures for statistical computing in Python. *Proceedings of the Python in Science Conference2010*.

[37] Harris C. R., Millman K. J., van der Walt S. J. (2020). Array programming with NumPy. *Nature*.

[38] Waskom M. (2021). seaborn: statistical data visualization. *Journal of Open Source Software*.

[39] Hunter J. D. (2007). Matplotlib: a 2D graphics environment. *Computing in Science & Engineering*.

[40] Parseliunas A., Paskauskas S., Kubiliute E., Vaitekunas J., Venskutonis D. (2021). Transcutaneous electric nerve stimulation reduces acute postoperative pain and analgesic use after open inguinal hernia surgery: a randomized, double-blind, placebo-controlled trial. *The Journal of Pain*.

[41] Bahreini M., Safaie A., Mirfazaelian H., Jalili M. (2020). How much change in pain score does really matter to patients?. *The American Journal of Emergency Medicine*.

[42] Cepeda S. M., Africano J. M., Polo R., Alcala R., Carr D. B. (2003). What decline in pain intensity is meaningful to patients with acute pain?. *Pain*.

[43] Kendrick D. B., Strout T. D. (2005). The minimum clinically significant difference in patient-assigned numeric scores for pain. *The American Journal of Emergency Medicine*.

[44] Olsen M. F., Bjerre E., Hansen M. D. (2017). Pain relief that matters to patients: systematic review of empirical studies assessing the minimum clinically important difference in acute pain. *BMC Medicine*.

[45] Bogduk N., Stojanovic M. (2020). Group data or categorical data for outcomes of pain treatment?. *Pain Medicine*.

[46] de Williams A. C., Davies H. T. O., Chadury Y. (2000). Simple pain rating scales hide complex idiosyncratic meanings. *Pain*.

[47] Kersten P., White P. J., Tennant A. (2014). Is the pain visual analogue scale linear and responsive to change? An exploration using rasch analysis. *PLoS One*.

[48] Nair A. S., Diwan S. (2020). Pain scores and statistical analysis—the conundrum. *Ain-Shams Journal of Anaesthesiology*.

[49] Kass R. E. (2011). Statistical inference: the big picture. *Statistical Science: A Review Journal of the Institute of Mathematical Statistics*.

[50] Conover W. J. (2009). Distribution-free methods in statistics. *WIREs Computational Statistics*.

[51] Divine G. W., Norton H. J., Barón A. E., Juarez-Colunga E. (2018). The wilcoxon–mann–whitney procedure fails as a test of medians. *The American Statistician*.

[52] Siegel S. (1956). *Nonparametric Statistics for the Behavioral Sciences*.

[53] Altman D. G. (1990). *Practical Statistics for Medical Research*.

[54] Friedman M. (1937). The use of ranks to avoid the assumption of normality implicit in the analysis of variance. *Journal of the American Statistical Association*.

[55] Spearman C. (1904). The proof and measurement of association between two things. *American Journal of Psychology*.

[56] Brown M. B., Forsythe A. B. (1974). Robust tests for the equality of variances. *Journal of the American Statistical Association*.

[57] Bland J. M. (2000). Statistics Notes: the odds ratio. *BMJ*.

[58] Howard C. W., Zou G., Morrow S. A., Fridman S., Racosta J. M. (2022). Wilcoxon-Mann-Whitney odds ratio: a statistical measure for ordinal outcomes such as EDSS. *Multiple Sclerosis and Related Disorders*.

[59] O’brien R. G. Exploiting the link between the wilcoxon-mann-whitney test and a simple odds statistic. *Paper presented at: Conference Proceedings*.

[60] Cumming T. B., Churilov L., Sena E. S. (2015). The missing medians: exclusion of ordinal data from meta-analyses. *PLoS One*.

[61] Perme M. P., Manevski D. (2018). Confidence intervals for the mann–whitney test. *Statistical Methods in Medical Research*.

[62] Pepe M. S. (2004). *The statistical evaluation of medical tests for classification and prediction*.

[63] Lautenbacher S., Peters J. H., Heesen M., Scheel J., Kunz M. (2017). Age changes in pain perception: a systematic-review and meta-analysis of age effects on pain and tolerance thresholds. *Neuroscience & Biobehavioral Reviews*.

[64] van Dijk J. F. M., Zaslansky R., van Boekel R. L. M. (2021). Postoperative pain and age: a retrospective cohort association study. *Anesthesiology*.

[65] Magaziner J., Simonsick E. M., Kashner T. M., Hebel J. R., Kenzora J. E. (1990). Predictors of functional recovery one year following hospital discharge for hip fracture: a prospective study. *Journal of Gerontology*.

[66] Johnson M. I., Paley C. A., Howe T. E., Sluka K. A. (2015). Transcutaneous electrical nerve stimulation for acute pain. *Cochrane Database of Systematic Reviews*.

[67] Simpson P. M., Fouche P. F., Thomas R. E., Bendall J. C. (2013). Transcutaneous electrical nerve stimulation for relieving acute pain in the prehospital setting. *European Journal of Emergency Medicine*.

[68] Cheing G. L. Y., Luk M. L. M. (2005). Transcutaneous electrical nerve stimulation for neuropathic pain. *Journal of Hand Surgery*.

[69] Law P., Cheing G. (2004). Optimal stimulation frequency of transcutaneous electrical nerve stimulation on people with knee osteoarthritis. *Journal of Rehabilitation Medicine*.

[70] Marchand S., Charest J., Li J., Chenard J.-R., Lavignolle B., Laurencelle L. (1993). Is TENS purely a placebo effect? A controlled study on chronic low back pain. *Pain*.

[71] Sluka K. A., Bjordal J. M., Marchand S., Rakel B. A. (2013). What makes transcutaneous electrical nerve stimulation work? Making sense of the mixed results in the clinical literature. *Physical Therapy*.

[72] Dias L. V., Cordeiro M. A., Schmidt de Sales R. (2021). Immediate analgesic effect of transcutaneous electrical nerve stimulation (TENS) and interferential current (IFC) on chronic low back pain: randomised placebo-controlled trial. *Journal of Bodywork and Movement Therapies*.

[73] Pivovarsky M. L. F., Gaideski F., Macedo R. M. d (2021). Immediate analgesic effect of two modes of transcutaneous electrical nerve stimulation on patients with chronic low back pain: a randomized controlled trial. *Einstein (São Paulo)*.

[74] Gozani S. (2016). Fixed-site high-frequency transcutaneous electrical nerve stimulation for treatment of chronic low back and lower extremity pain. *Journal of Pain Research*.

[75] Tan Z., Dong F., Wu L., Feng Y., Zhang M., Zhang F. (2023). Transcutaneous electrical nerve stimulation (TENS) alleviates brain ischemic injury by regulating neuronal oxidative stress, pyroptosis, and mitophagy. *Mediators of Inflammation*.

[76] Vance C. G. T., Radhakrishnan R., Skyba D. A., Sluka K. A. (2007). Transcutaneous electrical nerve stimulation at both high and low frequencies reduces primary hyperalgesia in rats with joint inflammation in a time-dependent manner. *Physical Therapy*.

[77] Correll D. (2017). Chronic postoperative pain: recent findings in understanding and management. *F1000Research*.

[78] Schug S. A., Bruce J. (2017). Risk stratification for the development of chronic postsurgical pain. *PAIN Reports*.

[79] Otto E., Culakova E., Meng S. (2022). Overview of Sankey flow diagrams: focusing on symptom trajectories in older adults with advanced cancer. *Journal of Geriatric Oncology*.

