# Supplementary figures and images for: Transcutaneous Electrical Nerve Stimulation Integrated into Pants for the Relief of Postoperative Pain in Hip Surgery Patients: A Randomized Trial
Source: Pain Res Manag. 2024 Jun 27;2024:6866549. doi: 10.1155/2024/6866549 (PMC11323988; doi:10.1155/2024/6866549)

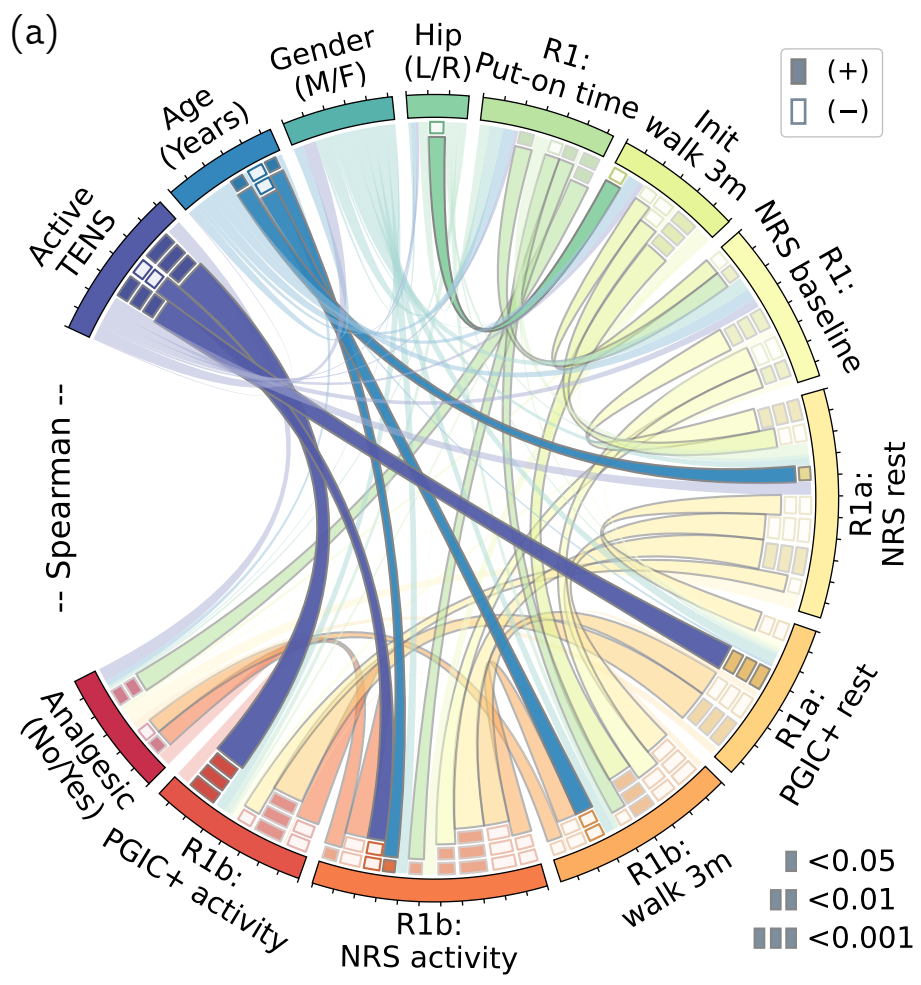

Supplement: Supplementary Materials — Figure S1: rank-order correlation (Spearman) between individual variables and outcomes measured during round 1 (a) and round 2 (b). The figure shows that pain ratings and impression of change are highly intercorrelated. Age, gender, and side of hip surgery have limited correlations with the outcome variables in round 1 and no significant correlation with the outcome variables in round 2. [file 6866549.f1.zip › Figure_S1a.pdf]

(b)

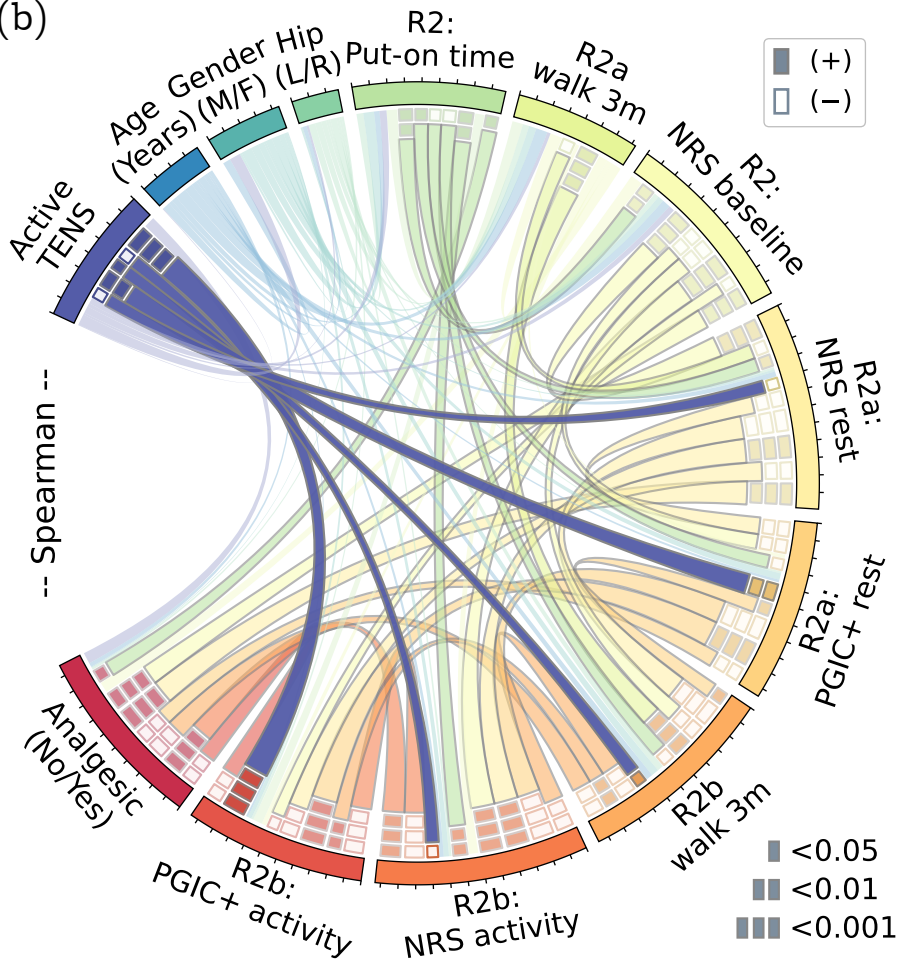

Supplement: Supplementary Materials — Figure S1: rank-order correlation (Spearman) between individual variables and outcomes measured during round 1 (a) and round 2 (b). The figure shows that pain ratings and impression of change are highly intercorrelated. Age, gender, and side of hip surgery have limited correlations with the outcome variables in round 1 and no significant correlation with the outcome variables in round 2. [file 6866549.f1.zip › Figure_S1b.pdf]
